# Supplementary material for: Magnon detection using a ferroic collinear multilayer spin valve
Source: Nat Commun. 2018 Mar 14;9:1089. doi: 10.1038/s41467-018-03485-5 (PMC5852167; doi:10.1038/s41467-018-03485-5)
Supplement: Supplementary file 1 — Supporting Information [file 41467_2018_3485_MOESM1_ESM.pdf]

SUPPLEMENTARY NOTE 1. SPIN PUMPING MEASUREMENTS

Spin transport from ferromagnetic insulators across antiferromagnetic insulators has been shown by a number of groups using $Y_3Fe_5O_{12}$ (YIG) as the ferro(i)magnet and 3d metal oxides for the antiferromagnetic insulator[1–5]. In particular using spin pumping by FMR excitation of the YIG, a spin current can be injected in the antiferromagnetic insulator.

SUPPLEMENTARY NOTE 1.1. Microwave absorption spectra

Supplementary Figure 1a-c shows the typical microwave absorption spectra obtained for sample A, B, and C. Here, the sample temperature is $T = 120\,\mathrm{K}$ and frequency and power of the applied microwave are $f = 4.5\,\mathrm{GHz}$ and $P = 23\,\mathrm{dBm}$, which corresponds to the values at which most of the characterization measurements were performed. For all samples ferromagnetic resonance appears in the same region ($H_{\mathrm{YIG}}^{\mathrm{FMR}} = 772 - 778$ Oe), which signifies equal properties of the YIG films and thus comparability of the samples all cut from a single wafer. The width of the absorption spectra, on the other hand, increases starting from sample A to sample C. This is reasonable considering that with increasing volume of both CoO and Co more angular momentum is absorbed, yielding an increased damping of the YIG precession and thus a wider absorption curve.

Note that by considering the absorption spectra in more detail, one can see that they are not composed of one single but several excited magnon modes present in the single crystal. This may yield the explanation, why spin current transmission and spin rectification signal exhibit slightly different resonance fields and line widths. The peak of the V_{sr} signal will be at the maximum-field of excited magnon modes in the 5 µm thick YIG crystal that couple to the Co magnetization via long-range dipolar exchange, whereas the peak of the V_{sc} signal will be at the field that corresponds to the maximum of excited modes that generate the spin current pumped into the CoO at the YIG|CoO interface. As the latter depends on the resonance at the interface while the former covers more of the bulk volume, the observed difference is not surprising.

SUPPLEMENTARY NOTE 1.2. Microwave absorption in the parallel and antiparallel state

Supplementary Figure 2a,b shows the absolute values of microwave absorption by sample A as a function of the magnetic field for parallel and antiparallel alignment of the YIG and Co magnetization at $T = 120\,\mathrm{K}$. The microwave frequency is $f = 4.5\,\mathrm{GHz}$, while the applied microwave power amounts to $P_{\mathrm{applied}} = 23\,\mathrm{dBm}$. Apparently, neither the maximum absorption nor the linewidth change significantly with the alignment state, which indicates a steady precession of the YIG magnetization independent of the alignment. This becomes even more clear when considering the relative differences between the absorption lines, which are plotted in Supplementary Figure 2c,d. A maximum difference of about 1 % is observed, which however is small as compared to the relative change of the amplitude of V_{sc} up to 120 %. Furthermore, such small changes can easily result from a slight mismatch of the applied magnetic field based on the used field step resolution.

SUPPLEMENTARY NOTE 1.3. FMR Spin pumping in sample B and C

In the main manuscript field-dependent voltage signals induced by microwave irradiation are exclusively presented for sample A. The frequency of the applied microwave is $f = 4.5\,\mathrm{GHz}$ and the absorbed microwave power (see Supplementary Figure 1) accounts for $P_{\mathrm{abs}} \approx 48\,\mathrm{mW}$. The voltage signal appears at ferromagnetic resonance of the YIG film and is composed of two distinct signals V_{sc} and V_{sr}, whose polarizations individually depend on the magnetization orientation of either YIG or Co. Furthermore, the magnitude of the spin current transmission signal V_{sc} depends on the relative alignment of YIG and Co. Supplementary Figure 3 and 4 show similar graphs obtained for sample B and C, respectively. For all cases the qualitatively same behavior is observed. The fact that the amplitude of V_{sc} in sample C is not significantly smaller as compared to sample B, as one might expect, could be explained by a non-trivial thickness dependence including an enhanced signal for a critical antiferromagnet thickness[1].

SUPPLEMENTARY NOTE 1.4. Pulse length dependence

In Supplementary Figure 5b the amplitude of V_{sr} is displayed as a function of the microwave pulse frequency/length f_{pulse}, revealing a rather weak dependence. If V_{sr} was resulting from an anomalous Nernst effect, one might expect a stronger impact of f_{pulse} considering slow thermal effects. An anomalous Hall effect induced spin rectification, on the other hand, should exhibit an instant response and not depend on the microwave pulse duration, matching the experimental observation. Nevertheless, such arguments must be treated with caution, especially taking into account the fact that the exact thermal properties of the samples are not perfectly well known.

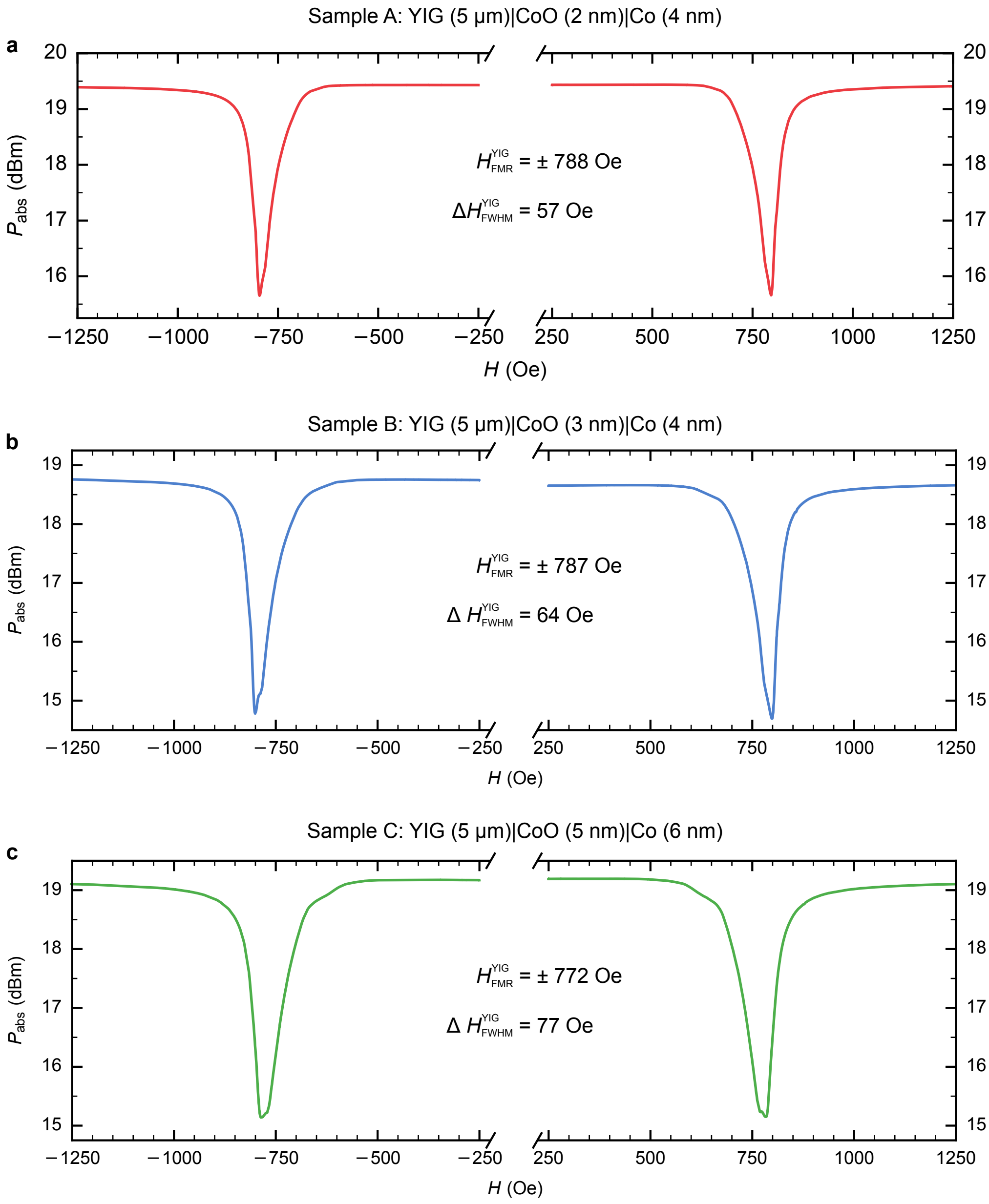

Supplementary Figure 1. **Microwave absorption spectroscopy.** Field dependent microwave absorption spectra of $Y_3Fe_5O_{12}$ (YIG) in (a) sample A [YIG (5 μm)|CoO (2 nm)|Co (4 nm)], (b) sample B [YIG (5 μm)|CoO (3 nm)|Co (4 nm)], and (c) sample C [YIG (5 μm)|CoO (5 nm)|Co (6 nm)] recorded at $T = 120\,K$. Frequency and power of the applied microwave are $f = 4.5\,GHz$ and $P = 23\,dBm$, respectively.

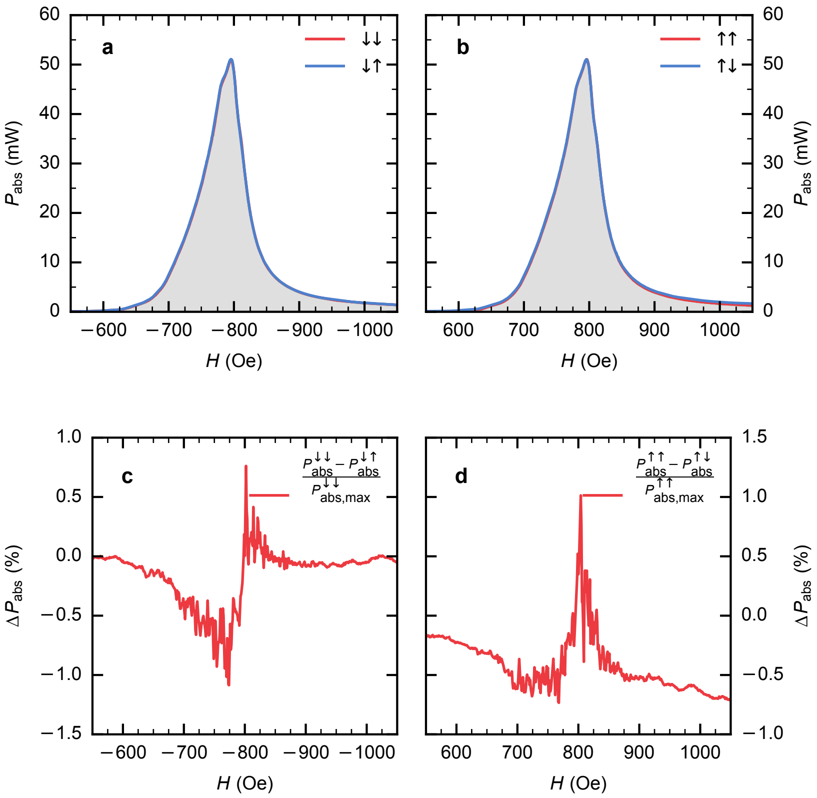

Supplementary Figure 2. **Microwave absorption spectroscopy for different alignment states.** (a),(b) Absorption spectra of $Y_3Fe_5O_{12}$ (YIG) in sample A [YIG (5 µm)|CoO (2 nm)|Co (4 nm)] obtained for microwave irradiation (f = 4.5 GHz, P_{applied} = 23 dBm) as a function of magnetic field for parallel and antiparallel alignment of YIG and Co magnetization, measured at T = 120 K. (c),(d) Difference of microwave absorption for parallel and antiparallel alignment of YIG and Co from (a),(b). Relative differences of up to 1 % are observed, which can result from small field shifts that originate from the uncertainty due to finite field step resolution used in the experiment.

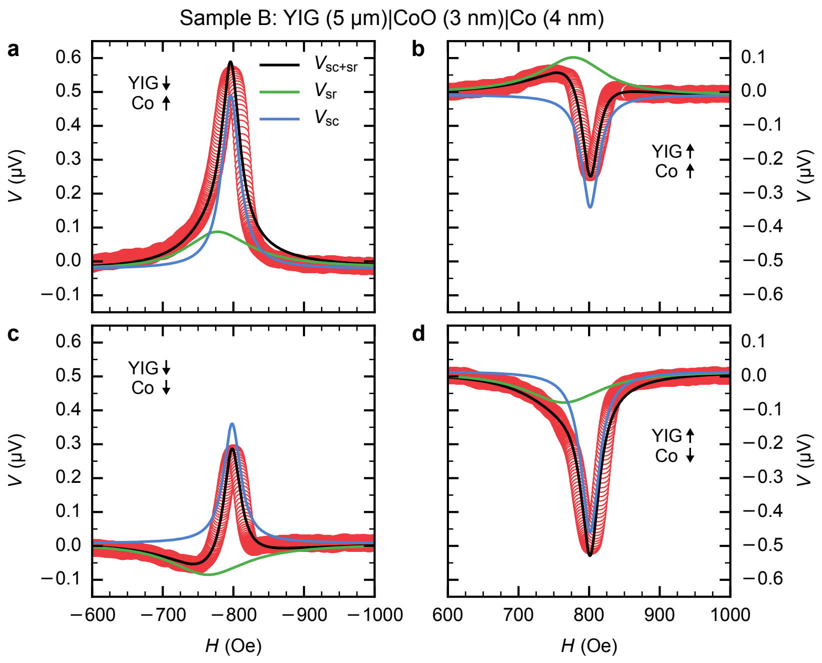

Supplementary Figure 3. **Alignment-dependent spin pumping in sample B.** Field-dependent voltage signals detected in sample B induced by microwave irradiation ($f = 4.5\,\mathrm{GHz}$, $P_{\mathrm{abs}} \approx 46\,\mathrm{mW}$, and $T = 120\,\mathrm{K}$).

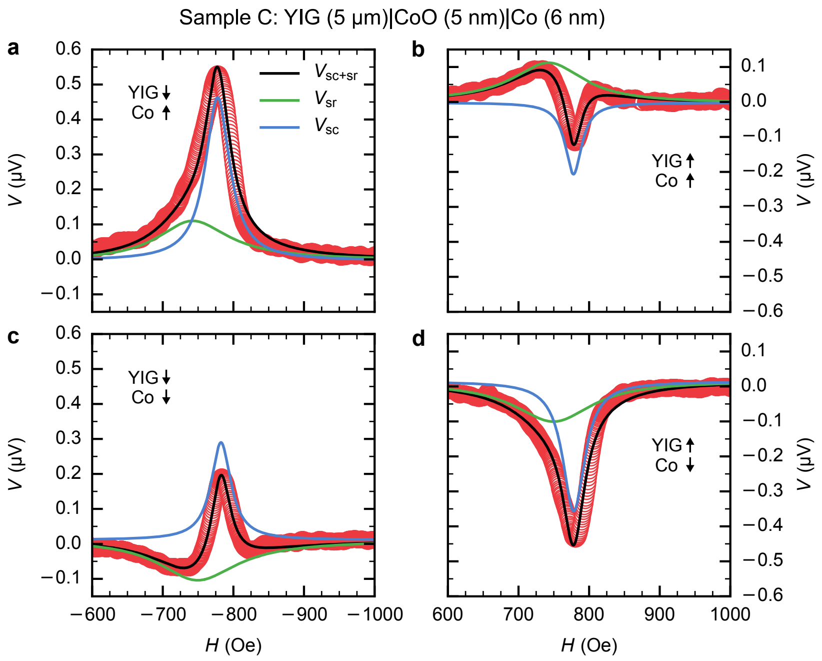

Supplementary Figure 4. **Alignment-dependent spin pumping in sample C.** Field-dependent voltage signals detected in sample C induced by microwave irradiation ($f = 4.5\,\mathrm{GHz}$, $P_{\mathrm{abs}} \approx 47\,\mathrm{mW}$, and $T = 120\,\mathrm{K}$).

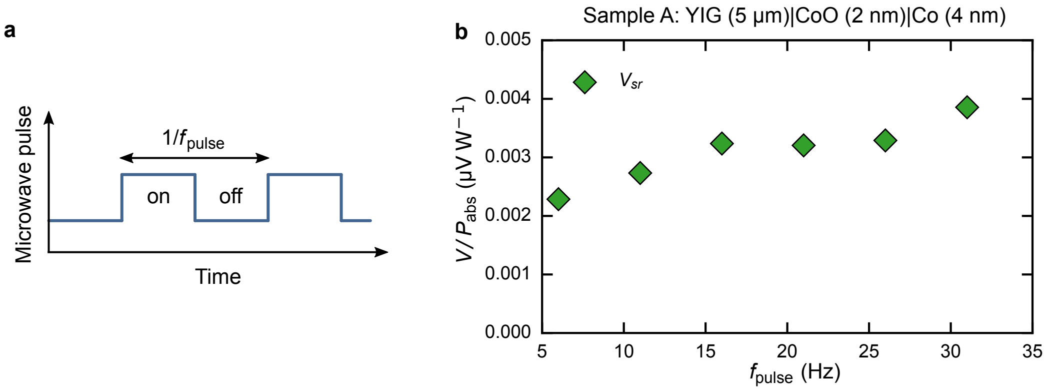

Supplementary Figure 5. **Pulse-length dependence of V_{sr} amplitude.** (a) Illustration of the pulse pattern. (b) Amplitude of V_{sr} as a function of the microwave pulse repetition frequency/pulse length.

SUPPLEMENTARY NOTE 2. SPIN-THERMOELECTRIC MEASUREMENTS

The large paramagnetic background of the $Gd_3Ga_5O_{12}$ substrate as well as the small volume of the Co film as compared to the YIG layer complicate the identification of the magnetic properties of the top Co layer with conventional methods (e.g. SQUID magnetometry). Therefore, spin thermoelectric measurements have been performed to observe the anomalous Nernst effect (ANE) of the Co film, since the ANE amplitude reflects the in-plane component of the Co magnetization. In Supplementary Figure 6 typical ANE hysteresis curves measured for sample A at different temperatures ($T = 60\,\mathrm{K}$, $160\,\mathrm{K}$ and $280\,\mathrm{K}$) are shown. At low temperatures large coercive fields $H_c^{\pm}$, a horizontal shift of the hysteresis by H_{ex} as well as an enhanced squareness $V_{\mathrm{ANE}}^{\mathrm{H=0}}/V_{\mathrm{ANE}}^{\mathrm{sat}}$ are observed. This behavior is due to the exchange bias effect, which besides a unidirectional anisotropy can introduce as well a uniaxial anisotropy term[6,7]. The latter can be explained by the presence of independent grains in the polycrystalline AFM. When sweeping the external field, the torques applied by these distinct grains lead to clockwise and counterclockwise reversal nucleation of domain walls in the FM, establishing a further energy barrier to overcome and thus increasing the switching field. With increasing temperature, the coercive and exchange bias field values decrease significantly, which is to be explained by the loss of the antiferromagnetic (AFM) order of the CoO film. The respective temperature dependent values are displayed in Supplementary Figure 7. Above $T \approx 250\,\mathrm{K}$ the exchange bias field induced by an additional anisotropy term vanishes and thus signifies the so-called blocking temperature T_B. The actual AFM phase transition of the CoO thin film occurs at $T_B \leq T_{\text{Néel}} \leq T_{\text{Néel}}^{\text{bulk}} = 291\,\mathrm{K}$.

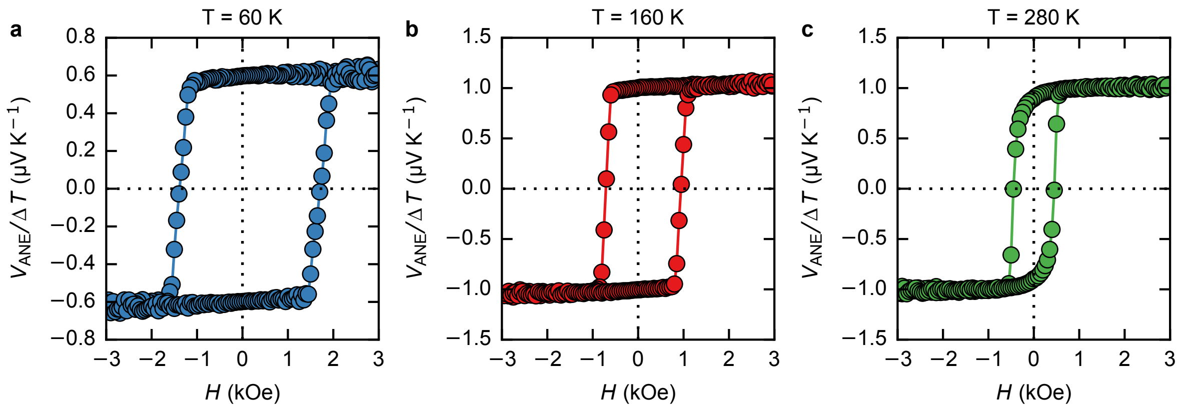

Supplementary Figure 6. Spin-thermoelectrics in $Y_3Fe_5O_{12}$ (YIG)|CoO|Co. Typical anomalous Nernst effect hysteresis curves measured in sample A [YIG (5 µm)|CoO (2 nm)|Co (4 nm)] measured for different temperatures (a) $T = 60\,\mathrm{K}$, (b) $T = 160\,\mathrm{K}$, and (c) $T = 280\,\mathrm{K}$.

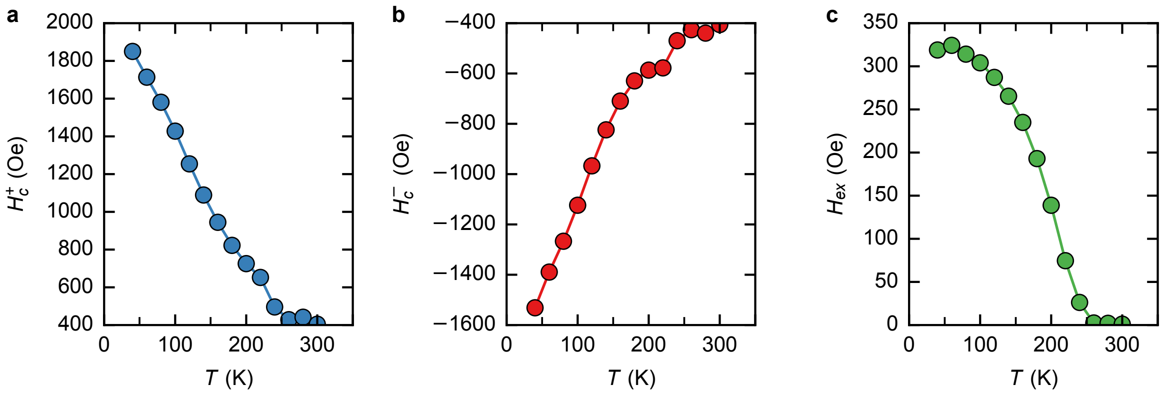

Supplementary Figure 7. **Magnetic properties of Co.** Positive and negative coercive fields as well as anisotropic exchange bias field of sample A [$Y_3Fe_5O_{12}$ (5 µm)|CoO (2 nm)|Co (4 nm)] as a function of temperature, extracted from anomalous Nernst effect hysteresis curves.

SUPPLEMENTARY NOTE 3. THEORY: PURE MAGNONIC SPIN VALVE

In the following, we investigate the effect of a pure magnonic spin valve. For that purpose, we consider an FM/AFM/FM-multilayer and vary the relative orientation of the magnetization of the two FM layers to demonstrate a higher efficiency of the transfer of spin current in the case of a parallel alignment of the magnetization of the two FMs.

We perform atomistic spin dynamic simulations by solving the Landau-Lifshitz-Gilbert (LLG) equation for each magnetic moment in each layer[8,9]. We use a Heisenberg model to describe the interactions of the magnetic system including exchange interaction with nearest neighbors and anisotropy. For simplification, we consider a layered AFM, in which the antiferromagnetic exchange coupling acts in z-direction, assuming $J_{\mathrm{AFM}} = -J_{\mathrm{FM}}$. The exchange interaction in the x-y-plane is ferromagnetic and equal to the exchange interaction of the FM layer. For the two interfaces, we assume antiferromagnetic interactions, again of same size $J_{\mathrm{IF}} = -J_{\mathrm{FM}}$. This system does not suffer from interface effects as, for example, a rotation of the magnetization of the AFM perpendicular to the FM magnetization due to spin-flop coupling. Furthermore, we consider in all layers an anisotropy with an easy-axis in z-direction with anisotropy constant $d_x = 0.01 J_{\mathrm{FM}}$. We simulate a cubic lattice with lattice constant a, a cross section of 8×8 magnetic moments, and assume that the lattice constant is the same in all layers. These simplifications will not affect the qualitative argument given by the simulations, they will only modify the quantitative value of the resulting spin currents.

In the simulations, we continuously excite a monochromatic spin wave with a given frequency ω and a wave vector $\boldsymbol{q}$ only in z-direction by a coherent precession of the 0th-layer of the system. This precession couples to the FM layer via exchange interaction. This method mimics the excitation by an oscillating external magnetic field. Its advantage is that we can control the amplitude of the excitation due to the strong exchange coupling.

The spin wave spectrum in the FM is obtained by solving the linearized LLG equation. The frequencies for a one-dimensional spin wave propagation with wave vector $\boldsymbol{q}$ in z-direction are:

$$\hbar\omega_{\mathrm{FM}} = 2d_x + 2J_{\mathrm{FM}}(1 - \cos(q_z a)) \tag{1}$$

Note that the magnetic moments in the FM will precess either clockwise or counter clockwise depending on the orientation of the magnetization, i. e. they have either right or left circular polarization. A spin wave carries magnetization with opposite sign than the saturation magnetization. During the propagation through the FM, its amplitude is damped depending on the damping constant α and its frequency ω_{FM}[10].

The excited spin wave propagates through the system and is partially transmitted into the AFM layer with constant frequency. The dispersion relation in the AFM differs from that of the FM and is given by:

$$\hbar\omega_{\mathrm{AFM}} = \sqrt{((2d_x - 2J_{\mathrm{AFM}})^2 - 4J_{\mathrm{AFM}}^2 \cos^2(q_z a)} \tag{2}$$

Due to the larger frequency gap, the spin waves from the FM excite in the AFM either a magnon for sufficiently high frequency or an evanescent mode below the frequency gap. In the latter case, the signal decays exponentially with distance from the interface[9]. AFMs are two-sublattice systems where magnons can have both of the two possible circular polarizations with the spins precessing either clockwise or counter clockwise. If a magnon propagates from the FM into the AFM, the polarization of the AFM mode follows the precession of

the FM. The amplitudes of the precession of the magnetic moments in the two sublattices of the AFM are not equal. As a result, magnetization is transferred through the AFM. By changing the magnetization direction of the FM, the spin transfer in the AFM is reversed and the precession of the sublattices is in the opposite direction.

The magnon propagation in an FM/AFM layer is shown exemplary in Supplementary Figure 8. We consider an FM with a magnetization aligned in x-direction. In the left part of Supplementary Figure 8, an excitation with a frequency $\omega \approx 0.5\mu_s/(\gamma J_{FM})$ is shown. This frequency is above the frequency gap for the given AFM and a spin wave is propagating through the AFM. The amplitudes of the precession in each sublattice are not equal and magnetic moment is transferred. The spin wave of the FM is only partially transmitted into the AFM and a large part of the signal is reflected at the interface. This can be seen by the interference of the incoming wave and the reflected part. The right part of Supplementary Figure 8 shows the situation for an evanescent mode with an excitation frequency $\omega \approx 0.1\mu_s/(\gamma J_{FM})$. In this case, the signal is exponential decaying in the antiferromagnet. The amplitude is oscillating in time due to the boundary conditions given by the FM, but the transferred angular momentum is negligible.

As a next step, we consider a trilayer consisting of an FM with 256 layers, an AFM of 10 layers and a second FM with 246 layers. We excite spin waves by the same method as before. Here, we vary the orientation of the magnetization of the two ferromagnetic layers. The resulting spin wave propagation for parallel alignment of the magnetization of the two FM's are shown exemplary in Supplementary Figure 9. In the left part, the propagation with a frequency $\omega \approx 0.5\mu_s/(\gamma J_{FM})$ above the frequency gap of the AFM is shown, whereas the right part illustrates the situation for an evanescent mode in the AFM. The excited spin wave propagates in both cases through the second interface and in the second ferromagnet a monochromatic spin wave is propagating through the system. Moreover, we observe that the transmitted signal is large compared to the transmission that was observed at the first interface. The antiferromagnet seems to act as a resonator and, therefore, the transmission into the second ferromagnet is very efficient.

The transmission of the spin wave is blocked if the magnetizations of the two ferromagnets are antiparallel to each other. As before we simulate frequencies above and below the frequency gap of the AFM. The results are shown in Supplementary Figure 10. Since the polarization of magnons in the first FM is not allowed in the second FM magnons cannot pass the second interface and no relevant spin transfer into the second FM occurs. Similar results are found for the evanescent modes in the AFM.

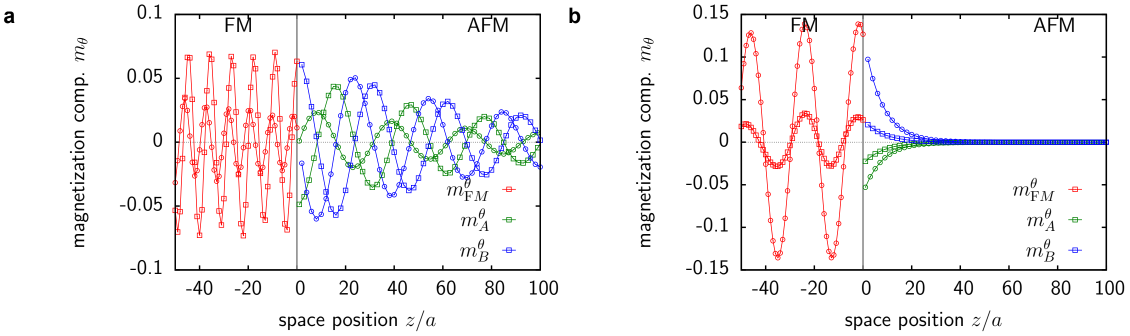

Supplementary Figure 8. **Magnon propagation in FM/AFM bilayers.** Spatial dependence of the y-components (square) and z-components (circle) of the magnetization for a spin wave in a two-layer system. (a) Excitation with frequency $\omega \approx 0.5\mu_{\mathrm{s}}/(\gamma J_{\mathrm{FM}})$ above the frequency gap in the AFM. (b) Excitation with frequency $\omega \approx 0.1\mu_{\mathrm{s}}/(\gamma J_{\mathrm{FM}})$ below the frequency gap in the AFM.

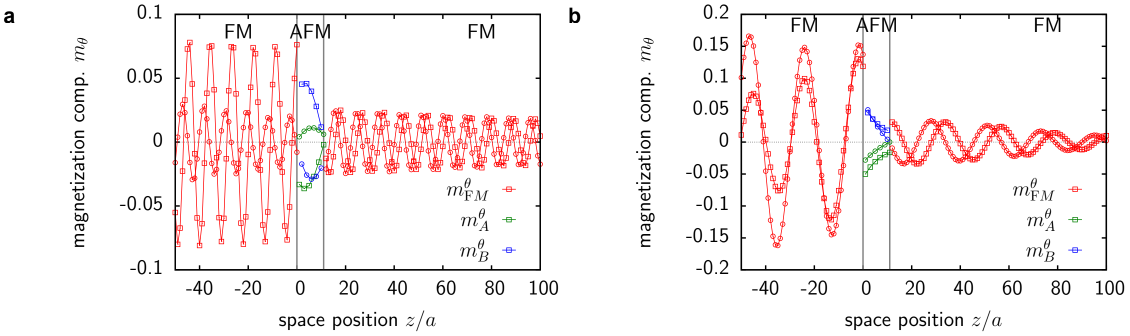

Supplementary Figure 9. **Magnon propagation in parallel FM/AFM/FM trilayers.** Spatial dependence of the y-components (square) and z-components (circle) of the magnetization for a spin wave in a FM-AFM-FM layer system with parallel alignment of the magnetization in the two FMs. (a) Excitation with frequency $\omega \approx 0.5\mu_{\mathrm{s}}/(\gamma J_{\mathrm{FM}})$ above the frequency gap in the AFM. (b) Excitation with frequency $\omega \approx 0.1\mu_{\mathrm{s}}/(\gamma J_{\mathrm{FM}})$ below the frequency gap in the AFM.

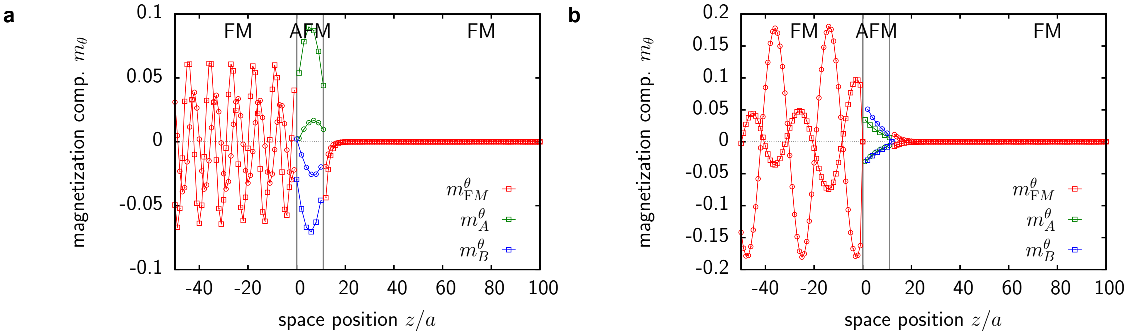

Supplementary Figure 10. **Magnon propagation in antiparallel FM/AFM/FM trilayers.** Spatial dependence of the y-components (square) and z-components (circle) of the magnetization for a spin wave in a FM-AFM-FM layer system with antiparallel alignment of the magnetization in the two FMs. (a) Excitation with frequency $\omega \approx 0.5\mu_{\mathrm{s}}/(\gamma J_{\mathrm{FM}})$ above the frequency gap in the AFM. (b) Excitation with frequency $\omega \approx 0.1\mu_{\mathrm{s}}/(\gamma J_{\mathrm{FM}})$ below the frequency gap in the AFM.

SUPPLEMENTARY NOTE 4. RAPID THERMAL ANNEALING OF YIG SURFACE & COO GROWTH

The single crystalline $Y_3Fe_5O_{12}$ (YIG) films used in this work were grown on a (111) oriented $Gd_3Ga_5O_{12}$ substrate by means of liquid phase epitaxy. Supplementary Figure 11a shows an atomic force microscopy (AFM) image of the surface of the respective YIG film after the growth procedure. To prepare the YIG samples for the subsequent deposition of CoO|Co bi-layers, further cleaning procedures as well as a rapid thermal annealing (RTA) process have been applied. For the latter, two samples were laid on top of each other such that the YIG films are in direct contact and afterwards heated up in an infrared furnace. The temperature was ramped up to 1173 K within 5 minutes, kept constant for 30 minutes, and finally ramped back to room temperature in approximately 25 minutes. This face-to-face RTA process yielded a smooth terrace-like YIG surface structure with a root mean square roughness of 1.11 Å, see Supplementary Figure 11b.

Subsequent X-ray spectroscopy data on YIG|CoO (d = 15 nm) reference samples, for which YIG was either treated or not treated by RTA, revealed that in the two cases the growth direction of the CoO layer is different. While the sample where the YIG surface was left as-is exclusively exhibits the CoO (200) peak, only the CoO (111) peak is visible for the RTA treated sample (see Supplementary Figure 12).

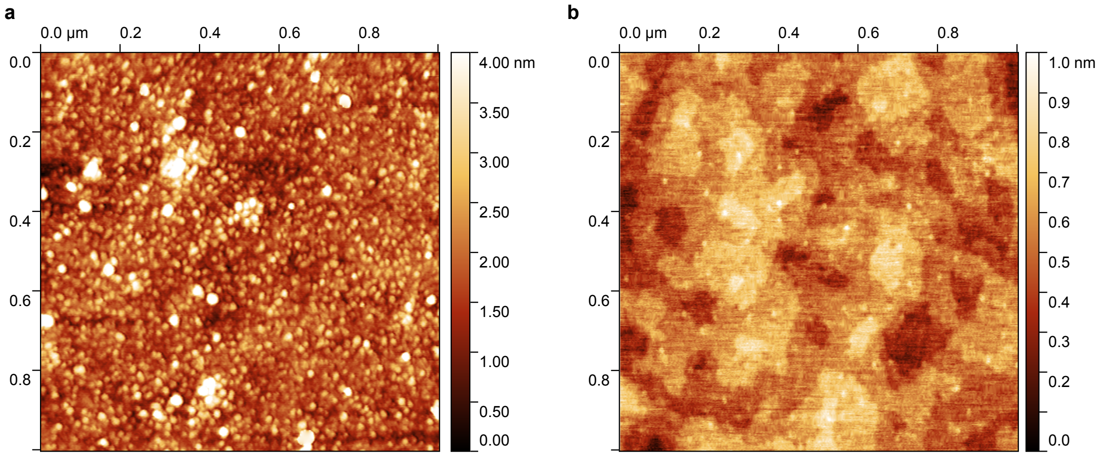

Supplementary Figure 11. Rapid thermal annealing of $Y_3Fe_5O_{12}$ (YIG). Atomic force microscopy data of liquid phase epitaxy grown YIG (a) after the growth procedure and (b) after treating the sample surface by means of rapid thermal annealing.

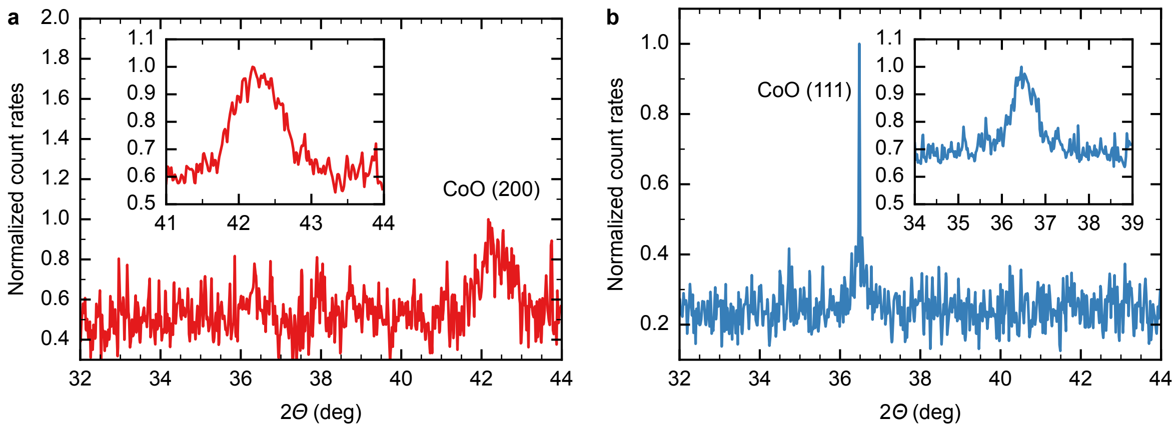

Supplementary Figure 12. **X-ray spectroscopy of $Y_3Fe_5O_{12}$ (YIG)|CoO.** X-ray diffraction data on YIG (5 µm)|CoO (15 nm) samples, which distinguish on whether YIG was treated or not by rapid thermal annealing. For (a) YIG as-is the CoO (200) peak appears, whereas (b) annealed samples exhibit the CoO (111) peak. The insets show more detailed scans of the individual peaks.

SUPPLEMENTARY REFERENCES

[1] Wang, H., Du, C., Hammel, P. C. & Yang, F. Antiferromagnonic spin transport from $Y_3Fe_5O_{12}$ into NiO. *Phys. Rev. Lett.* **113**, 097202 (2014).

[2] Hahn, C. *et al.* Conduction of spin currents through insulating antiferromagnetic oxides. *Europhys. Lett.* **108**, 57005 (2014).

[3] Moriyama, T. *et al.* Anti-damping spin transfer torque through epitaxial nickel oxide. *Appl. Phys. Lett.* **106**, 162406 (2015).

[4] Lin, W., Chen, K., Zhang, S. & Chien, C. Enhancement of thermally injected spin current through an antiferromagnetic insulator. *Phys. Rev. Lett.* **116**, 186601 (2016).

[5] Qiu, Z. *et al.* Spin-current probe for phase transition in an insulator. *Nat. Commun.* **7**, 12670 (2016).

[6] Ambrose, T., Sommer, R. & Chien, C. L. Angular dependence of exchange coupling in ferromagnet/antiferromagnet bilayers. *Phys. Rev. B* **56**, 83 (1997).

[7] Stiles, M. & McMichael, R. Model for exchange bias in polycrystalline ferromagnet-antiferromagnet bilayers. *Phys. Rev. B* **59**, 3722 (1999).

[8] Nowak, U. *Handbook of Magnetism and Advanced Magnetic Materials*, vol. 2, chap. Classical Spin-Models (John Wiley & Sons, New York, 2007).

[9] Khymyn, R., Lisenkov, I., Tiberkevich, V. S., Slavin, A. N. & Ivanov, B. A. Transformation of spin current by antiferromagnetic insulators. *Phys. Rev. B* **93**, 224421 (2016).

[10] Ritzmann, U., Hinzke, D. & Nowak, U. Propagation of thermally induced magnonic spin currents. *Phys. Rev. B* **89**, 024409 (2014).
